# TFP-YOLO: Obstacle and Traffic Sign Detection for Assisting Visually Impaired Pedestrians

**DOI:** 10.3390/s25185879

**Published:** 2025-09-19

**Authors:** Zhiwei Zheng, Jin Cheng, Fanghua Jin

**Affiliations:** School of Science, Beijing Information Science and Technology University, Beijing 100192, China; 2023021114@bistu.edu.cn (Z.Z.); 2023020471@bistu.edu.cn (F.J.)

**Keywords:** computer vision, machine guide dog, object detection, YOLOv8

## Abstract

With the increasing demand for intelligent mobility assistance among the visually impaired, machine guide dogs based on computer vision have emerged as an effective alternative to traditional guide dogs, owing to their flexible deployment and scalability. To enhance their visual perception capabilities in complex urban environments, this paper proposes an improved YOLOv8-based detection algorithm, termed TFP-YOLO, designed to recognize traffic signs such as traffic lights and crosswalks, as well as small obstacle objects including pedestrians and bicycles, thereby improving the target detection performance of machine guide dogs in complex road scenarios. The proposed algorithm incorporates a Triplet Attention mechanism into the backbone network to strengthen the perception of key regions, and integrates a Triple Feature Encoding (TFE) module to achieve collaborative extraction of both local and global features. Additionally, a P2 detection head is introduced to improve the accuracy of small object detection, particularly for traffic lights. Furthermore, the WIoU loss function is adopted to enhance training stability and the model’s generalization capability. Experimental results demonstrate that the proposed algorithm achieves a detection accuracy of 93.9% and a precision of 90.2%, while reducing the number of parameters by 17.2%. These improvements significantly enhance the perception performance of machine guide dogs in identifying traffic information and obstacles, providing strong technical support for subsequent path planning and embedded deployment, and demonstrating considerable practical application value.

## 1. Introduction

According to global statistics, over 2.2 billion people worldwide suffer from some form of visual impairment [1]. In China alone, the visually impaired population is approximately 17.31 million, making it the country with the largest number of blind individuals. Notably, 23.5% of this group are adolescents or young to middle-aged adults. This substantial demographic faces an urgent demand for safe and efficient mobility assistance tools. At present, most navigation methods rely on smartphones for global route planning. While such systems can offer general directional guidance, they fall short in providing timely and detailed local navigation during movement, particularly when encountering dynamic obstacles such as pedestrians, bicycles, and vehicles, or static traffic elements such as crosswalks and traffic lights. This limitation poses serious challenges to the efficiency and safety of travel for the visually impaired.

**Figure 1:**
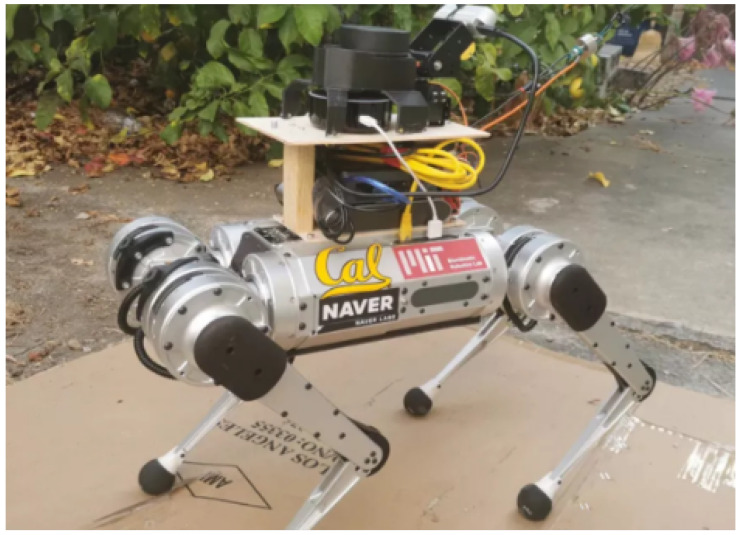
Schematic diagram of robot guide dog.

With the rapid advancement of robotic assistance technologies and intelligent perception algorithms, machine guide dogs are increasingly regarded as an ideal alternative to traditional guide dogs. These systems are becoming a crucial component in the mobility solutions available to visually impaired individuals. Compared to real guide dogs, which are costly to train and difficult to scale, quadruped robotic guide dogs based on intelligent navigation and environmental perception offer advantages such as high replicability and flexible deployment. In recent years, Xiao et al. [2] proposed a robot guide dog system based on the hybrid physical interaction of the Mini Cheetah quadruped robot and a traction rope. The robot guide dog system can guide blind people to move safely in narrow environments through a rope with variable tension and relaxation, demonstrating the feasibility and effectiveness of robots in assisting blind people to navigate in real scenarios, as shown in Figure 1. However, ensuring the reliable operation of such systems in complex environments hinges on the ability to accurately and efficiently perceive typical obstacles and traffic cues in real time.

Therefore, constructing a visual perception system capable of road target detection is essential for the effective operation of guide robots. On the one hand, such a system enables accurate detection of common obstacles and traffic signs in road environments, thereby preventing visually impaired users from deviating from the intended path due to occlusions or ambiguous route information. On the other hand, it provides a reliable environmental awareness foundation for subsequent local path planning and dynamic obstacle avoidance. Vision-based object detection modules have thus become a core component in achieving safe, stable, and intelligent guidance for machine guide dogs.

The detection of typical road obstacles and traffic signs has long been a research focus in the field of computer vision. Currently, mainstream approaches in object detection utilize a combination of radar, ultrasonic sensors, and vision-based systems. Among these, visual detection offers richer scene information, lower cost, and easier deployment compared to alternative sensing technologies. Visual detection typically involves capturing scenes using cameras and applying algorithms to identify objects of interest within the images.

At present, there are two detection methods used in target detection tasks: traditional detection methods and deep learning detection methods. Traditional target detection algorithms use sliding windows and manually extracted features [3,4]. The Regionlets [5] detection model generates multiple local regions by modeling each region of the object, and trains these regions through a support vector machine (SVM). This model can better cope with the morphological changes and complex backgrounds of the object. The Deformable Part Model (DPM) [6] detection model extracts local features from the image to represent each part, and then applies these features to the part model through convolution operations. The core idea of DPM was later further developed in deep learning models. Traditional detection methods have a lot of redundant calculations, a slow running speed, poor robustness in complex environments, and other problems, making it difficult for them to achieve satisfactory detection results. In contrast, Ross Girshick et al. [7] proposed the application of deep convolutional networks to the field of target detection and designed a new network architecture R-CNN to improve the accuracy of object detection and compared it with the traditional target detection algorithm on the VOC 2010 dataset. The detection accuracies of multiple categories such as bike, car, and person are higher than those of traditional detection methods, and the average accuracy is also higher than that of traditional methods. Deep learning algorithms do not require manual feature extraction and have strong anti-interference ability, so they are widely used in the field of target detection.

Traditional methods usually rely on manually designed feature extractors and classifiers, facing problems such as complex backgrounds, occlusion, and scale changes. By introducing target detection technology based on deep learning, breakthrough progress has been made. Detection algorithms based on deep learning are mainly divided into two-stage and single-stage detection algorithms. The two-stage detection algorithm obtains the detection result by extracting candidate boxes and performing secondary correction. Representative algorithms include R-CNN [8], Fast R-CNN [9], Faster-RCNN [10], etc. Single-stage algorithms can complete positioning and classification at one time. Representative algorithms include the SSD series of algorithms [11,12,13] and the YOLO series of algorithms [14]. In the past few years, researchers have developed the target detection framework from a two-stage to a one-stage framework.

The YOLO [15,16] algorithm adopts a single-stage target detection method, which divides the entire image into grid cells and predicts multiple bounding boxes and category probabilities in each cell to achieve rapid detection of traffic signs. Compared with traditional methods, the YOLO [17] algorithm has higher detection speed and accuracy.

The core idea of the algorithm is to learn feature representation from the original image through a deep convolutional neural network (CNN) and then use the predictor to generate bounding boxes and category probabilities. YOLOv8 adopts the deep neural network structure of Darknet, combined with feature extraction at different levels, to effectively capture the shape, texture, and contextual information of traffic signs. In addition, the YOLOv8 algorithm also introduces a series of optimization strategies, such as multi-scale training, data enhancement, and loss function optimization, to further improve the performance of obstacle and traffic sign detection.

Existing object detection methods, particularly those based on the YOLO series, have shown remarkable performance in real-time applications. YOLOv8, with its efficient Darknet backbone and multi-scale feature extraction, achieves a strong balance of speed and accuracy. However, it faces limitations in the context of machine guide dog applications. First, YOLOv8 struggles with small target detection (e.g., traffic lights smaller than 8×8 pixels), as deeper network layers lose fine-grained details due to downsampling. Second, blurred-edge features, such as crosswalks under occlusion or varying lighting, are often misdetected due to weak edge representations. Third, the Complete IoU (CIoU) loss function in YOLOv8 overemphasizes low-quality samples, reducing robustness in complex scenes with scale variations and boundary ambiguities. These shortcomings hinder reliable navigation assistance in diverse urban environments, necessitating targeted improvements for visually impaired applications.

To address these challenges, this paper designs a new feature fusion network architecture that combines local and global feature information to obtain more accurate feature maps. Compared with the original network structure, it has fewer parameters and higher average accuracy.

The contributions of this paper are summarized as follows, and are quantitatively verified on both public and custom datasets:

1 A lightweight Triplet Attention module is introduced into the backbone network. It captures cross-dimensional interactions to enhance the correlation among local regions and the interactions between feature channels. This mechanism significantly improves the network’s focus on indistinct features with blurred edges, such as crosswalks, resulting in an approximately 4.6% improvement in the recall rate for this category of objects.
2 We design a multi-scale feature enhancement module, termed Triple Feature Encoding (TFE). It fuses spatial information from three different feature map resolutions (large, medium, and small). This structure facilitates the extraction of fine-grained details from small objects and reduces background noise interference. Working in concert with the P2 detection head, it achieves a 5.2% increase in average precision (AP) for small objects such as traffic lights.
3 A P2 detection head is employed to construct a four-head multi-scale detection architecture. It extracts lower-level features from higher-resolution feature maps, which aids in identifying small-scale targets. This design lowers the model’s effective detection size limit from 8 × 8 pixels to 4 × 4 pixels, significantly enhancing the perception of very small objects. It collaborates with other detection heads to effectively handle objects of varying scales.
4 The Complete IoU (CIoU) loss function is replaced with Wise-IoU v3 (WIoU). Its dynamic focusing mechanism addresses the challenges of blurred boundaries and scale variations by enhancing the focus on hard samples (e.g., small traffic lights) while reducing the emphasis on low-quality samples. This replacement ultimately improves the mean average precision (mAP@0.5) by 4.1% while reducing the number of parameters by 17.2%, achieving a better trade-off between accuracy and efficiency.

## 2. Related Work

Recent advancements in object detection, particularly within the YOLO series, have focused on improving accuracy for small targets and complex urban scenes, which are critical for applications like machine guide dogs. These systems require real-time detection of obstacles and traffic signs to ensure safe navigation for visually impaired users.

Early improvements to YOLO models emphasized using attention mechanisms to enhance feature representation. For instance, Yan et al. [18] integrated the Squeeze-and-Excitation (SE) Block into YOLOv5, achieving a 1.44% mAP improvement by prioritizing salient features. Similarly, Li et al. [19] incorporated SE Net and CBAM into YOLOv3’s backbone, boosting mAP by up to 8.50% through channel-wise importance learning. Ma et al. [20] introduced the Feature Select Module (FSM) in the neck layer of YOLOv3, YOLOv4, and YOLOv5-L, reducing noise in feature fusion and improving performance by 0.60% to 1.50%. Ju et al. [21] proposed AFFAM for YOLOv3, combining global and spatial attention for multi-scale feature fusion, yielding mAP gains of 5.08% to 7.41% on datasets like KITTI.

More recent works have targeted small object detection in traffic scenarios, directly relevant to urban navigation challenges. For example, the ETSR-YOLO model [22] enhanced YOLO for multi-scale traffic sign detection, improving robustness in complex environments. TSD-YOLO [23] introduced a Space-to-Depth module to handle scale variations in traffic signs, addressing missed detections. DP-YOLO [24] optimized YOLOv8s for small traffic signs by reducing parameters while boosting accuracy. SOD-YOLOv8 [25] specifically improved YOLOv8 for small objects in traffic scenes, incorporating optimizations for urban drone imagery. CAS-YOLOv8 [26] enhanced remote sensing object detection with contextual attention, showing promise for urban small targets.

Despite these advances, existing models often increase parameter complexity or compromise real-time performance, limiting their deployment in resource-constrained guide dog systems. Moreover, few address the unique needs of visually impaired navigation, such as detecting blurred-edge features (e.g., crosswalks) alongside small targets (e.g., traffic lights) in varied urban conditions. This study builds on YOLOv8’s efficiency, introducing targeted improvements to achieve a balance of accuracy, speed, and lightweight design for machine guide dog applications.

While robust perception is fundamental, a complete machine guide dog system also requires advanced path planning and navigation algorithms to ensure safe and efficient guidance. Traditional global planners such as A* [27] and Dijkstra’s algorithm [28] perform well in static environments but lack real-time reactivity to unknown obstacles. In contrast, local planners like the Dynamic Window Approach (DWA) [29] offer high reactivity but may suffer from local minima and suboptimal global performance.

To address these limitations, hybrid approaches that integrate global and local planning have emerged. A notable example is the BRRT*-DWA framework with Adaptive Monte Carlo Localization (AMCL) proposed by Ayalew et al. [30], which combines bidirectional rapidly exploring random tree star (BRRT*) for global path generation with DWA for real-time obstacle avoidance in dynamic environments.

Crucially, the performance of such navigation systems highly depends on the accuracy of perceptual inputs. Our work enhances this pipeline by providing a highly accurate visual perception module that reliably detects obstacles (e.g., pedestrians, vehicles) and traffic elements (e.g., crosswalks, traffic lights). These outputs enable robust downstream path planning and localization, ultimately improving the safety and effectiveness of the machine guide dog system.

## 3. TFP-YOLO Method

### 3.1. TFP-YOLO Model

**Figure 2:**
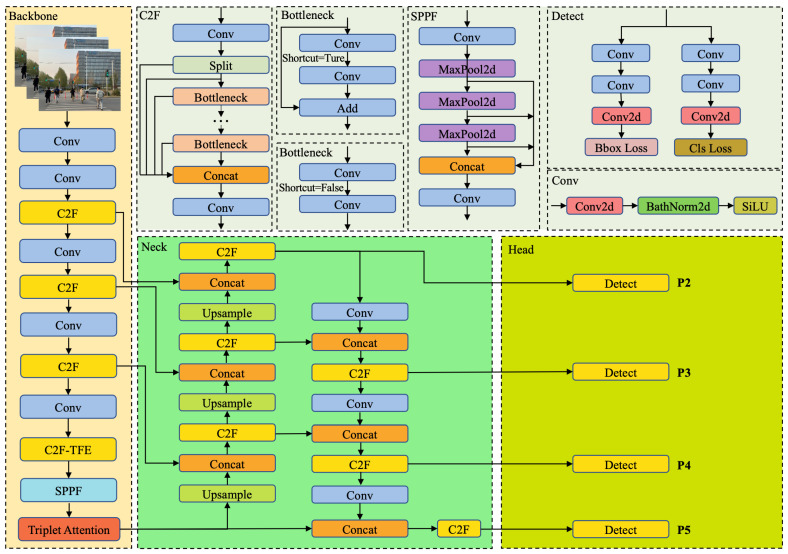
Architecture of proposed TFP-YOLO. The backbone network integrates a series of Conv and C2F modules to extract initial features, enhanced by a Triplet Attention mechanism. The neck module employs C2F and SPPF structures, incorporating Bottleneck and Concat operations to fuse multi-scale features, with upsampling and shortcut connections for improved information flow. The detection head utilizes multiple detect layers (P2–P5) to predict bounding boxes and class probabilities, optimized by Bbox Loss and Cls Loss.

To enable effective detection of obstacles and traffic signs encountered during the navigation of machine guide dogs, the network model must achieve a balance between low parameter complexity and high mean average precision (mAP). In this work, we propose improvements to the original YOLOv8 architecture by introducing modifications to its backbone, neck, and head components. These enhancements are designed to increase detection accuracy while reducing the overall model size. The structure of the improved network is illustrated in Figure 2.

### 3.2. Triplet Attention Mechanism

**Figure 3:**
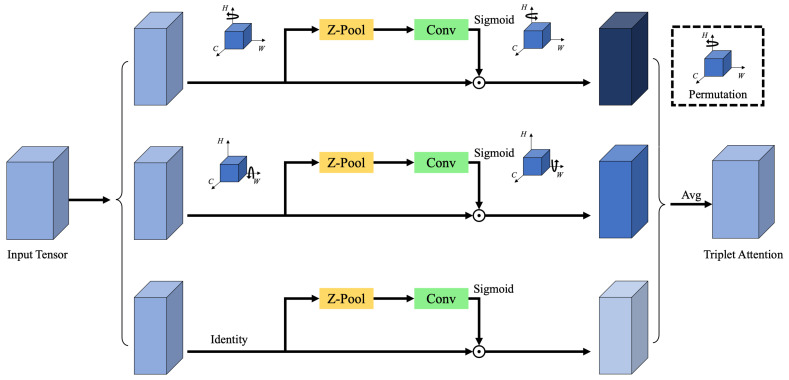
Diagram of triplet attention.

To improve detection accuracy for challenging targets such as crosswalks, which often exhibit unclear or fragmented edge features, the Triplet Attention mechanism is embedded at the end of the backbone network. This attention module enhances the network’s sensitivity to fine-grained spatial patterns, particularly in cases where edge information is weak. Triplet Attention is a three-branch module that receives an input tensor and outputs a feature tensor of the same shape. By applying directional transformations across its branches, the module allows edge features—such as those of crosswalk lines—to be captured from multiple orientations, thereby improving the network’s robustness in such detection tasks. The structural diagram of the Triplet Attention mechanism is shown in Figure 3.

Given an input feature tensor $x\in R^{C\times H\times W}$, the Triplet Attention module processes it through three independent branches.

#### 3.2.1. Branch 1 (Height–Channel Interaction)

The input *x* is first permuted to shape (H×W×C), then rotated $90^{\circ }$ counterclockwise along the height *H* axis, producing ${\hat{x}}_{1}$. This transformation promotes interaction between the height and channel dimensions. The rotated tensor is then passed through a Z-Pool layer, which reduces the height dimension to 2. The resulting tensor ${\hat{x}}_{1}$ is fed into a standard convolutional layer with kernel size k×k, generating a feature map of shape (1×H×C). A Batch Normalization layer follows, and the output is passed through a sigmoid activation function to produce attention weights. These weights are then applied to the rotated feature map, which is subsequently rotated $90^{\circ }$ clockwise to restore the original spatial configuration.

#### 3.2.2. Branch 2 (Width–Channel Interaction)

Similarly, the input *x* is rotated $90^{\circ }$ counterclockwise along the width *W* axis, resulting in a tensor ${\hat{x}}_{2}$ with shape (H×C×W). Z-Pool reduces the height dimension, generating ${\hat{x}}_{2}^{*}\in R^{\left(2\times C\times W\right)}$, which is then processed by a convolutional layer (kernel size k×k) to produce an attention map of shape (1×C×W). This output is normalized and activated using BatchNorm and a sigmoid function, respectively, and the attention weights are applied to ${\hat{x}}_{2}^{*}$. Finally, the tensor is rotated $90^{\circ }$ clockwise to match the original input orientation.

#### 3.2.3. Branch 3 (Spatial Attention)

In the third branch, the channel dimension of the input *x* is pooled via Z-Pool, resulting in ${\hat{x}}_{3}\in R^{\left(2\times H\times W\right)}$. This is passed through a k×k convolutional layer followed by Batch Normalization and a sigmoid activation, producing a spatial attention map that is applied directly to the input *x*.

**Figure 4:**
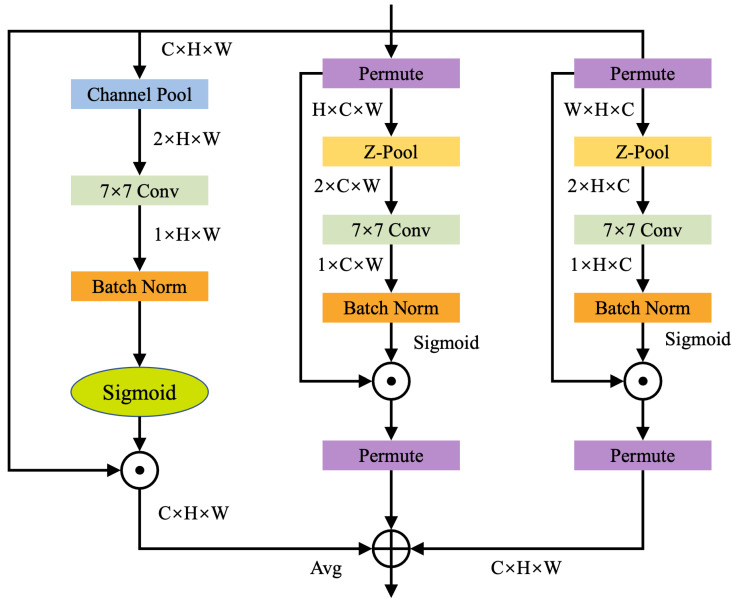
Network topology diagram.

Each branch produces an attention-weighted feature map of shape (C×H×W), and these three outputs are fused via element-wise averaging to obtain the final attention-enhanced feature map. The overall structure of this mechanism is illustrated in Figure 4.

The Z-Pool operation is a critical component in all three branches. It applies max pooling and average pooling along the channel dimension of the input tensor, then concatenates the results. This operation not only reduces feature depth, thereby lowering computational complexity, but also retains rich semantic information from the feature maps. The process is formally defined as:

(1) $$\mathrm{Z-Pool}\left(x\right)=\left[\mathrm{MaxPool}(x),\mathrm{AvgPool}(x)\right]$$

The formula expression for the Triplet Attention mechanism is:

(2) $$y=\frac{1}{3}\left(\bar{{\hat{x}}_{1}\delta \left(\psi_{1}\left({\hat{x}}_{1}^{*}\right)\right)}+\bar{{\hat{x}}_{2}\delta \left(\psi_{2}\left({\hat{x}}_{2}^{*}\right)\right)}+x\delta \left(\psi_{3}\left({\hat{x}}_{3}\right)\right)\right)$$

where δ denotes the sigmoid activation function and $\psi_{1}$, $\psi_{2}$, and $\psi_{3}$ represent the standard 2D convolutional layers of the three branches in Triplet Attention, with a kernel size of *k*. After simplifying (2), y becomes:

(3) $$y=\frac{1}{3}\left(\bar{{\hat{x}}_{1}\omega_{1}}+\bar{{\hat{x}}_{2}\omega_{2}}+x\omega_{3}\right)=\frac{1}{3}\left(\bar{y_{1}}+\bar{y_{2}}+y_{3}\right)$$

where $\omega_{1}$, $\omega_{2}$, and $\omega_{3}$ are the three attention weights computed in the Triplet Attention mechanism. In (3), $\bar{y_{1}}$ and $\bar{y_{2}}$ indicate that the original features have been rotated $90^{\circ }$ clockwise.

### 3.3. Triple Feature Encoding Module

**Figure 5:**
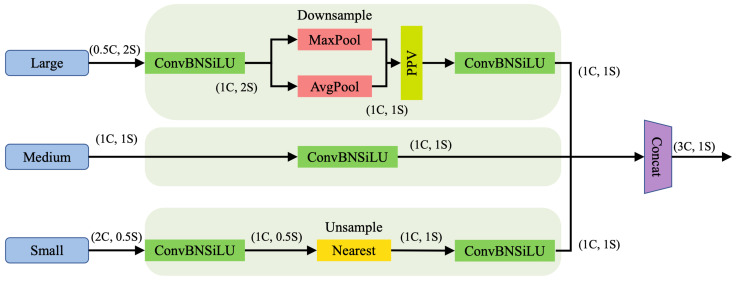
The structure of TFE module. C represents the number of channels and S represents the feature map size. Each Triple Feature Encoder module uses three feature maps of different sizes as input.

The feature map sizes and resolutions of each layer in the backbone network vary. Traditional fusion mechanisms, such as the standard FPN, typically only upsample small-sized feature maps and then fuse them with the features of the previous layer, often neglecting the rich detailed information contained in large-sized feature maps. In contrast, the proposed TFE module explicitly splits and processes feature maps from three different scales (large, medium, and small). Specifically, it incorporates large-scale feature maps and performs downsampling on them, while upsampling the small-scale feature maps. This process unifies their spatial dimensions to match the medium-scale feature map. By doing so, the TFE module effectively integrates both high-resolution details from shallow layers and high-level semantic information from deep layers, thereby significantly enhancing the extraction of feature information for small targets such as traffic lights. The structure of the TFE module is shown in Figure 5.

The formula is as follows:

(4) $$F_{\mathrm{TFE}}=\mathrm{Concat}\left(F_{l},F_{m},F_{s}\right)$$

where $F_{\mathrm{TFE}}$ represents the feature map output by the TFE module. $F_{l}$, $F_{m}$, and $F_{s}$ represent the large-, medium-, and small-sized feature maps, respectively. $F_{l}$, $F_{m}$, and $F_{s}$ are combined to form $F_{\mathrm{TFE}}$, which has the same resolution as $F_{m}$ but three times the number of channels.

### 3.4. P2 Detection Head

After the 640×640 input image passes through the backbone and neck, the head of YOLOv8 sets three detection heads P3, P4, and P5 by default, corresponding to feature maps of 80×80, 40×40, and 20×20, respectively, for detecting large, medium, and small targets. However, the original YOLOv8n can only detect targets larger than 8×8 pixels, and it struggles to effectively identify key obstacles such as traffic lights and traffic signs with smaller sizes.

**Figure 6:**
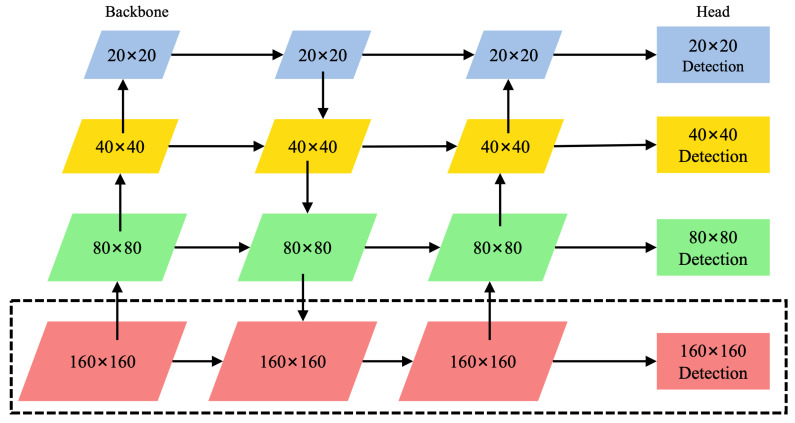
Add P2 detection head.

To improve the detection ability of small targets, this paper adds a 160×160 detection head to the P2 layer. As shown in Figure 6, the model introduces an upsampling operation based on the 80×80 feature map, expands it to 160×160, and fuses it with the corresponding scale features in the backbone, while adding a downsampling path to match the structure. Finally, four detection heads are constructed to jointly complete multi-scale target detection, which significantly enhances the model’s recognition ability for small targets. The specific detection scales are shown in Table 1.

### 3.5. Wisev3-IoU Loss Function

The original CIoU loss function used in YOLOv8n tends to overemphasize low-quality samples in the training set due to factors such as distance and aspect ratio, which can impair the model’s generalization performance. To enhance detection performance, this paper introduces the WIoU loss function with a dynamic focusing mechanism for bounding box regression. This method mitigates the excessive punishment of geometric factors on the model by reducing the competitiveness of high-quality boxes and weakening the harmful gradients generated by low-quality samples, thereby improving the model’s generalization and localization capabilities.

WIoU is defined as:

(5) $$\beta =\frac{L_{\mathrm{IoU}}}{{\bar{L}}_{\mathrm{IoU}}}\in \left[0,+\infty \right)$$

(6) $$L_{\mathrm{IoU}}=1-\mathrm{IoU}$$

(7) $$K_{\mathrm{WIoU}}=\mu R_{\mathrm{WIoU}}L_{\mathrm{IoU}}$$

(8) $$R_{\mathrm{WIoU}}=\exp\left(\frac{{\left(x-x_{\mathrm{gt}}\right)}^{2}+{\left(y-y_{\mathrm{gt}}\right)}^{2}}{{\left(W_{g}^{2}+H_{g}^{2}\right)}^{*}}\right)$$

(9) $$\mu =\frac{\beta }{\sigma \alpha^{\left(\beta -\sigma \right)}}$$

where β denotes the outlier degree, IoU is the intersection-over-union between the predicted and ground truth boxes, $L_{\mathrm{IoU}}$ is the loss, and ${\bar{L}}_{\mathrm{IoU}}$ is its moving average normalization factor. $K_{\mathrm{WIoU}}$ is the WIoU loss, μ is the gradient gain, σ and α are hyperparameters, and $R_{\mathrm{WIoU}}$ represents the normalized center distance between the predicted and ground truth boxes. Coordinates *x*, *y*, and $x_{\mathrm{gt}}$, $y_{\mathrm{gt}}$ correspond to the predicted and true box centers, while $W_{g}$ and $H_{g}$ are the width and height of their minimum enclosing box. The asterisk (*) indicates that $W_{g}$ and $H_{g}$ are excluded from gradient computation to avoid adverse training effects.

**Figure 7:**
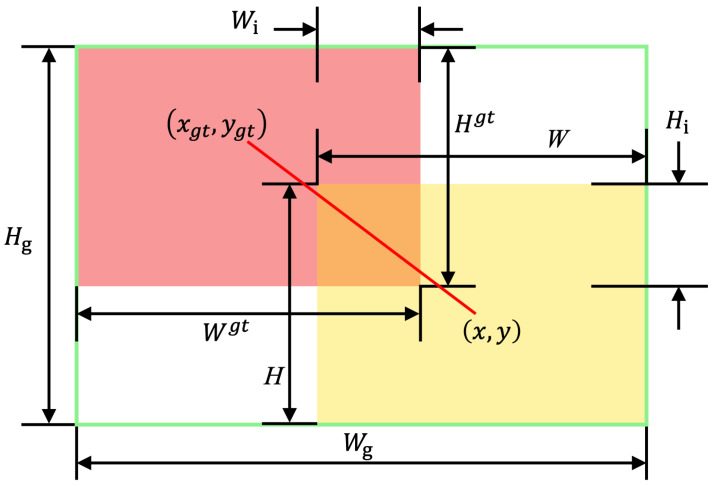
Description of WIoU.

Smaller β indicates higher-quality anchor boxes; assigning them lower gradient gains suppresses harmful gradients from outliers. When β=σ, μ=1, granting maximum gradient gain. To preserve this strategy early in training, ${\bar{L}}_{\mathrm{IoU}}$ is initialized to 1, and a momentum term $m=1-\sqrt[{tn}]{0.05}$ (with *t* as epoch and *n* as batch size) is used to delay its convergence. In later stages, WIoU reduces gradients for low-quality anchors and shifts attention toward mid-quality ones to enhance localization. Hyperparameters α and σ are set to 1.9 and 3, respectively. The parameter dynamics are illustrated in Figure 7.

### 3.6. Module Synergy Analysis

The four proposed components—Triplet Attention (TA), Triple Feature Encoding (TFE), P2 detection head, and Wise-IoU (WIoU) loss—work synergistically to address the core challenges of small and blurred object detection.

#### 3.6.1. TA and TFE: Enhanced Feature Representation

The TA mechanism amplifies crucial spatial and channel-wise features, enhancing fine-grained patterns essential for identifying indistinct targets like crosswalks. The TFE module directly benefits from this refined input. By concatenating multi-scale features, it effectively fuses high-resolution details with deep semantic context. This ensures the detection heads receive feature maps rich in salient information for accurate detection.

#### 3.6.2. TFE and P2 Head: Precision for Small Targets

The TFE module’s emphasis on large-scale feature maps is perfectly complemented by the high-resolution (160×160) P2 detection head. This combination creates a dedicated pathway for small objects. TFE provides a robust, multi-scale feature set, mitigating information loss, while the P2 head leverages this to achieve precise localization and classification of targets as small as 4×4 pixels, a task challenging for standard heads.

#### 3.6.3. WIoU Loss: Robust Learning

The high-quality features produced by TA and TFE provide clearer signals for hard samples (small, occluded, or blurred objects). The WIoU loss capitalizes on this by dynamically assigning higher gradient gains to lower-quality samples (higher β), focusing the learning process in these challenging cases. This synergy between superior feature encoding and a focused loss function significantly enhances model robustness and generalization.

#### 3.6.4. Holistic Integration

This cohesive design ensures TFP-YOLO operates as an integrated system, not merely a collection of parts. Each component amplifies the others’ effectiveness, resulting in superior detection performance critical for machine guide dog navigation.

## 4. Experiment and Result Analysis

### 4.1. Dataset

This study utilizes both publicly available datasets and a custom-made dataset. The publicly available dataset consists of a subset of the RoadSign dataset that meets the requirements for small object detection, totaling 877 images, including 701 training images and 176 validation images. These images cover four common types of traffic signs: crosswalk, traffic light, stop, and speed-limit.

The TSDD dataset [31], a Chinese traffic sign dataset released by the National Natural Science Foundation of China, comprises 10,000 street scene images containing nearly 30,000 traffic signs of various types. The images were collected under diverse conditions including different times, weather, lighting, and motion blur, and are all original images. From these, 2000 images were selected as the test set.

The GTSDB dataset [32] consists of street scene images captured under various conditions in Germany, with a total of 900 images and over 2000 annotated objects. The signs are categorized into mandatory signs, prohibitory signs, danger signs, and others. From this dataset, 600 images were chosen as the test set.

**Figure 8:**
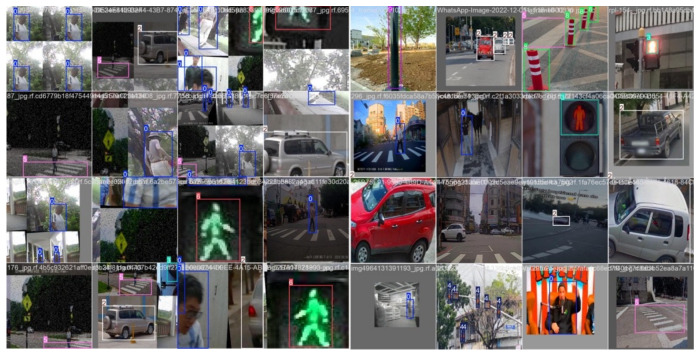
Dataset partial sample.

The custom dataset was collected by a camera on sidewalks in Beijing. After recording videos, frames were extracted using a Python script, with an image resolution of 640 × 640, covering various weather conditions such as sunny, rainy, and snowy days. The dataset includes eight types of typical obstacles and traffic signs, including pedestrians, cars, bicycles, crosswalks, traffic lights, poles, and traffic cones. The data is divided into a training set (8929 images), a validation set (1078 images), and a test set in a 0.8:0.1:0.1 ratio, with the latter two sets being mutually independent. Some samples and annotations are shown in Figure 8.

### 4.2. Experimental Environment and Evaluation Indicators

#### 4.2.1. Experimental Environment Setting

This experiment was conducted on an Ubuntu 22.04 platform using an NVIDIA RTX 4090 device with 24 GB of video memory. The training environment consisted of Python 3.10, PyTorch 2.1.0, and CUDA 12.1.

#### 4.2.2. Experiment Parameter Settings

During training for urban street scene recognition, the input sample size of the model was converted to 640 × 640, the batch size was set to 16, the SGD optimizer was used, the epoch was set to 200, the learning rate was 0.01, the momentum was 0.937, and the weight decay coefficient was 0.0005. Data augmentation techniques included random horizontal flipping, random scaling, mosaic augmentation, and mixup. The specific parameter settings are shown in Table 2.

This paper evaluates the model using precision (*P*), recall (*R*), mean average precision (mAP), mAP50, parameter count, and FPS, where higher values indicate better performance. The metrics are calculated as:

(10) $$P=\frac{TP}{TP+FP}$$

(11) $$R=\frac{TP}{TP+FN}$$

(12) $$AP=\int_{0}^{1}P\left(r\right)dr$$

(13) $$mAP=\frac{1}{n}\sum_{i=1}^{n}AP_{i}$$

where TP, FP, and FN represent true positives, false positives, and false negatives, respectively. mAP50 computes the average AP at IoU = 0.5, while FPS measures processing speed in frames per second.

## 5. Result and Discussion

### 5.1. Comparison Experiment of Different Weight Versions of YOLOv8

Pre-trained weights provide valuable prior knowledge for object detection models through transfer learning, accelerating convergence and improving performance. Their effectiveness varies across different weighting schemes and datasets. Table 3 shows the detection performance of YOLOv8 with different weights for obstacles and traffic signs.

Table 3 shows that the weighting model affects detection accuracy, speed, and parameter count. YOLOv8n offers the best balance with minimal parameters (3.01M) and the fastest speed, making it ideal for guide robot perception. We therefore select YOLOv8n as our base model.

### 5.2. Attention Mechanism Comparison Experiment

To validate the superiority of our Triplet Attention module, we compared it against CBAM, SE, CA, and EMA mechanisms in YOLOv8. As shown in Table 4, Triplet Attention outperforms others by capturing cross-dimensional interactions more efficiently, enhancing local feature relevance and channel relationships while maintaining lightweight design.

Table 4 compares different attention mechanisms in YOLOv8n. While EMA shows good performance, Triplet Attention achieves superior results with 91.4% mAP0.5, 91.3% precision, and 87.2% recall.

### 5.3. Loss Function Comparison Experiment

To validate the improved YOLOv8n’s detection performance, we compared various IoU metrics (Wise-IoUv3, CIoU, DIoU, EIoU, SIoU) as shown in Table 5. For vehicle detection tasks with imbalanced categories (e.g., pedestrians) and small targets (e.g., traffic lights), Wise-IoUv3 addresses these challenges through its dynamic weighting mechanism. It assigns larger weights to small categories and neglected regions, preventing model bias toward dominant categories while improving small object detection.

Table 5 shows that the enhanced YOLOv8n (with Triplet Attention, TFE, and P2) achieves 92.1% mAP0.5 (90.8% P, 86.3% R) using CIoU. Among tested WIoUv3 variants, the configuration with α=1.9 and σ=3 yields optimal performance, 93.9% mAP0.5 (90.2% P, 89.8% R), surpassing other loss functions.

### 5.4. Analysis of Object Detection Results

To validate the detection capability of the proposed algorithm model in complex road scenarios, experimental verification was conducted on two public datasets. Table 6 and Table 7 present the comparative detection results between the YOLOv8 model and the proposed model on the validation sets of these two public datasets. Experimental results demonstrate that on the TSDD dataset, the proposed model achieves improvements of 5.3 percentage points in mAP50 and 7.6 percentage points in mAP50:95. Similarly, on the GTSDB dataset, it shows improvements of 5.7 percentage points in mAP50 and 3.4 percentage points in mAP50:95.

### 5.5. Ablation Experiment

To validate the effectiveness of each module in enhancing the Yolov8n network, ablation experiments were conducted using Yolov8n as the baseline. Metrics included average precision, accuracy, recall, and parameter count, as shown in Table 8 (where ✓ indicates inclusion of a module). Results show that each added module improved performance to varying degrees. The Wise-IoUv3 module achieved the highest AP gain of 1.7% without increasing parameters, demonstrating its effectiveness in improving bounding box localization for varied object shapes and scales. The P2 module enhanced AP by 1.1%, particularly benefiting small target detection such as traffic lights. Although combining P2 and Wise-IoUv3 yielded slightly lower AP than Wise-IoUv3 alone, it reduced parameters to 2.74 M. The full integration of Triplet Attention, TFE, P2, and Wise-IoUv3 boosted Yolov8n’s accuracy to 93.9%, with 2.49 M parameters and 182 FPS.

The results presented in Table 6 not only demonstrate the performance improvements achieved by each proposed module but also reveal their distinct effect sizes and underlying mechanisms.

The Triplet Attention (TA) module boosts performance (+1.6% mAP) by enhancing cross-dimensional spatial–channel interactions, which is critical for recognizing objects with weak textual cues (e.g., crosswalks), as reflected in the increased recall.

The TFE module’s primary role is to aggregate and preserve multi-scale features, supplying richer representations for the detection heads. Its effect is most evident when combined with the P2 head.

The P2 detection head provides a substantial gain (+1.1% mAP) by leveraging high-resolution (160 × 160) features. This is decisive for small objects (e.g., traffic lights), as it drastically improves localization precision for targets below 8 × 8 pixels.

The Wise-IoU v3 (WIoU) loss brings the largest individual improvement (+1.7% mAP) by introducing a dynamic focusing mechanism that suppresses gradients from low-quality examples, thereby improving generalization and robustness.

The full model’s performance (93.9% mAP) demonstrates clear synergy: TFE provides multi-scale features, TA refines their representation, P2 detects small objects precisely, and WIoU ensures stable training. This integration achieves an optimal accuracy–efficiency balance.

### 5.6. Comparison of Different Models

We compared mainstream models including YOLOv5n, YOLOv6n, and YOLOv10n in terms of parameter count, average precision, accuracy, and FPS to evaluate the performance of the proposed model, as summarized in Table 9.

As shown in Table 9, although YOLOv6n slightly surpasses YOLOv8n in average accuracy, it suffers from significantly lower FPS. Models such as YOLOv5n, YOLOv10n, and YOLOv11n achieve comparable accuracy but with reduced speed. YOLOv7 and Faster R-CNN not only have much larger parameter counts but also perform worse in both accuracy and FPS.

Overall, YOLOv8n offers a better balance of accuracy, speed, and model size than other unmodified models. The proposed improved model further reduces parameters by 0.52M, boosts accuracy by 4.1%, and achieves higher precision and FPS, validating the effectiveness of our approach.

### 5.7. Algorithm Verification

**Figure 9:**
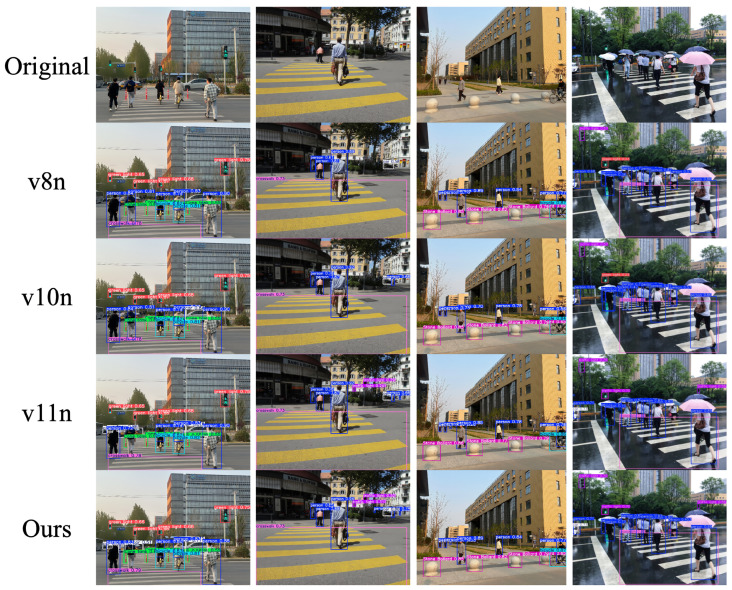
Comparison of different methods.

The improved algorithm was compared with YOLOv8n, YOLOv10n, and YOLOv11n—top performers in average accuracy—to evaluate detection on typical obstacles for visually impaired users, including crosswalks and traffic signs under various scenarios and weather conditions. Results are shown in Figure 9.

The improved algorithm reduces false negatives for small targets like traffic lights and lowers false positives for blurred-edge targets compared to the other three algorithms. This enhances obstacle and traffic sign detection for guide dogs in complex environments, improving local path planning accuracy and reliability and laying a solid foundation for future research.

### 5.8. Implementation of YOLO Framework on NVIDIA Jetson Orin Nano Super

Today, single-board computers like the Nvidia Jetson Orin Nano Super are gaining popularity for edge computing applications, including artificial intelligence and deep learning. The Jetson Orin Nano Super, featuring a 6-core Arm^®^ Cortex^®^-A78AE v8.2 64-bit CPU, a 1024-core NVIDIA Ampere architecture GPU with 32 Tensor Cores, an 8 GB 128-bit LPDDR5 RAM offering 102 GB/s bandwidth, and comprehensive high-speed I/O support, delivers outstanding AI computational performance up to 67 TOPS with remarkable power efficiency. In this study, we leverage the benefits of this embedded edge computing device specifically for model reasoning, coupled with an Intel RealSense D435i camera to form a comprehensive perception system. Given that model training demands more powerful computing resources than the testing process, we perform dedicated model training tasks on a GPU-equipped workstation in the cloud. The generated weight files are deployed on the edge device, where the Jetson Orin Nano Super processes real-time visual data from the D435i camera to perform efficient obstacle and traffic sign detection in various environmental conditions.

**Figure 10:**
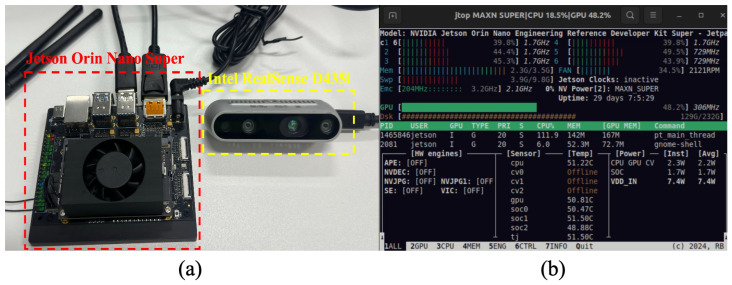
Implementation of the obstacle detection system on NVIDIA Jetson platform: (**a**) Jetson Orin Nano Super developer kit and Intel RealSense D435i depth camera; (**b**) real-time system performance monitoring.

To validate the practical deployment of the proposed system, we established a complete experimental setup utilizing the Jetson Orin Nano Super as the central processing unit and the Intel RealSense D435i camera as the visual perception module. In this configuration, the D435i camera serves as the “eyes” of the machine guide dog, continuously capturing RGB video streams at 1280 × 720 resolution with a frame rate of 30 FPS. The Jetson Orin Nano Super functions as the main controller, executing real-time inference using the optimized YOLO model. The physical implementation of this hardware system is illustrated in Figure 10.

The experimental results demonstrate that the integrated system achieves outstanding performance metrics: a detection precision of 94.2% and a recall rate of 91.6%, indicating high accuracy and reliability in obstacle and traffic sign recognition. The system maintains an average processing throughput of 28.7 FPS, ensuring smooth real-time operation. The per-frame inference latency remains below 50 ms, providing responsive feedback for navigation assistance. Regarding resource utilization, the GPU occupancy is approximately 42%, with memory consumption of 3.1 GB, indicating efficient resource management. The total power consumption is controlled at 8.7 W, demonstrating the energy efficiency of the edge deployment. All performance indicators meet the expected targets for real-world machine guide dog applications, validating the effectiveness of the proposed hardware–software co-design approach. The detailed performance metrics are systematically summarized in Table 10.

## 6. Conclusions

To address the challenges of detecting small targets (e.g., traffic lights) and blurred-edge features (e.g., crosswalks) commonly encountered by guide dogs in complex environments, this paper proposes an improved YOLOv8-based detection model. Triplet Attention is integrated into the backbone to enhance local feature and channel modeling. The TFE module is introduced to fuse multi-scale and spatial information, boosting small target extraction. A P2 detection head further improves detection of small objects, while the WIoU loss optimizes bounding box regression, improving robustness against low-quality samples. Experiments demonstrate that the improved model achieves 93.9% mAP and 90.2% precision with a 17.2% reduction in parameters. Future work will focus on lightweight strategies like pruning and quantization to enable efficient deployment on guide dog systems in real-world scenarios.

## Figures and Tables

**Table 1:** Detection head dimension table.

Detection Head	P5	P4	P3	P2 (New)
Feature map size	20×20	40×40	80×80	160×160
Target size	≥32×32	≥16×16	≥8×8	≥4×4

**Table 2:** Training parameter settings.

Parameter Name	Parameter Value
Input size	640×640
Initial learning rate	0.01
Minimum learning rate	0.0001
Epochs	200
Batch size	16
Optimizer	SGD
Momentum	0.937
Weight decay	0.0005
Number of threads	8
Ratio	0.75

**Table 3:** Detection results of different weight models of Yolov8.

Model	mAP0.5/%	P/%	R/%	Params (M)
YOLOv8n	89.8	87.7	85.2	3.01
YOLOv8s	92.4	91.4	89.1	11.21
YOLOv8m	93.1	92.8	88.8	23.64
YOLOv8l	92.9	92.6	87.9	42.13
YOLOv8x	93.6	91.5	89.3	66.46

**Table 4:** Comparison of different attention mechanisms.

Model (YOLOv8n)	mAP0.5/%	P/%	R/%
CBAM	90.7	89.6	86.2
SE	90.6	89.4	85.6
CA	90.7	89.7	86.2
EMA	91.2	90.3	84.8
Triplet Attention	91.4	91.3	87.2

**Table 5:** Comparison of different loss functions.

Model (YOLOv8n + TA + T + P)	mAP0.5/%	P/%	R/%
CIoU	92.1	90.8	86.3
DIoU	91.7	90.0	86.0
EIoU	92.0	90.5	86.3
SIoU	91.8	90.6	86.1
WIoUv3 (α=1.4, σ=5)	93.8	89.9	87.2
WIoUv3 (α=1.6, σ=4)	93.4	89.2	87.4
WIoUv3 (α=1.9, σ=3)	93.9	90.2	89.8

**Table 6:** Comparison of model performance before and after improvement on the TSDD dataset.

Category	YOLOv8n		Proposed Model	
	**mAP50/%**	**mAP50:95/%**	**mAP50/%**	**mAP50:95/%**
Traffic sign	86.3	45.2	91.6	52.8
All	86.3	45.2	91.6	52.8

**Table 7:** Comparison of model performance before and after improvement on the GTSDB dataset.

Category	YOLOv8n		Proposed Model	
	**mAP50/%**	**mAP50:95/%**	**mAP50/%**	**mAP50:95/%**
Mandatory	82.8	61.9	88.5	62.3
Prohibitory	91.5	73.0	96.8	75.5
Danger	91.2	63.9	97.1	72.0
Others	80.4	59.0	86.3	61.6
All	86.5	64.5	92.2	67.9

**Table 8:** Ablation experiment.

Model	TA	TFE	P2	WIoU	mAP/%	P/%	R/%	Params (M)	FPS
v8n					89.8	87.7	85.2	3.01	176
1	✓				91.4	91.3	87.2	3.05	170
2		✓			90.6	87.8	84.9	2.91	179
3			✓		90.9	88.1	85.4	3.01	184
4				✓	91.5	89.8	87.8	3.01	176
5	✓	✓			91.6	91.8	86.4	2.49	173
6			✓	✓	91.2	89.7	85.8	2.74	190
7	✓	✓	✓		92.8	90.1	88.0	2.49	188
8	✓	✓	✓	✓	93.9	90.2	89.8	2.49	182

**Table 9:** Comparison of different models.

Model	mAP@0.5/%	P/%	Params (M)	FLOPs (G)	FPS
YOLOv5n	89.4	88.6	2.51	7.4	162
YOLOv6n	90.0	87.6	4.23	48.9	74
YOLOv7	89.6	89.9	37.2	19.2	142
YOLOv8n	89.8	87.7	3.01	18.0	176
YOLOv10n	88.6	86.8	2.71	22.7	128
YOLOv11n	89.0	85.0	2.58	23.5	120
Faster R-CNN	79.8	83.7	41.2	38.2	58
MobileNet-SSD	78.6	81.2	12.13	24.7	86
NanoDet	78.6	80.2	0.92	1.9	231
Ours	93.9	90.2	2.49	12.8	182

**Table 10:** Performance metrics of the deployed system on Jetson Orin Nano Super.

Performance Metric	Value	Unit	Target
Detection Precision	94.2	%	≥90.0
Recall Rate	91.6	%	≥90.0
Average FPS	28.7	FPS	≥25.0
Per-frame Latency	<50	ms	≤50
GPU Utilization	42.0	%	≤60
Memory Consumption	3.1	GB	≤4.0
Power Consumption	8.7	W	≤10.0

## Data Availability

The dataset of this article can be downloaded at https://github.com/zzw0709/TFP-YOLO (accessed on 16 August 2025). The source code of TFP-YOLO can be obtained from the corresponding author.
